# Diversity and antibiotic susceptibility of autochthonous dairy enterococci isolates: are they safe candidates for autochthonous starter cultures?

**DOI:** 10.3389/fmicb.2015.00954

**Published:** 2015-09-09

**Authors:** Amarela Terzić-Vidojević, Katarina Veljović, Jelena Begović, Brankica Filipić, Dušanka Popović, Maja Tolinački, Marija Miljković, Milan Kojić, Nataša Golić

**Affiliations:** ^1^Laboratory for Molecular Microbiology, Institute of Molecular Genetics and Genetic Engineering, University of BelgradeBelgrade, Serbia; ^2^Faculty of Pharmacy, University of BelgradeBelgrade, Serbia

**Keywords:** *Enterococcus* sp., artisan dairy products, diversity, antibiotic susceptibility, virulence

## Abstract

Enterococci represent the most controversial group of dairy bacteria. They are found to be the main constituent of many traditional Mediterranean dairy products and contribute to their characteristic taste and flavor. On the other hand, during the last 50 years antibiotic-resistant enterococci have emerged as leading causes of nosocomial infections worldwide. The aim of this study was to determine the diversity, technological properties, antibiotic susceptibility and virulence traits of 636 enterococci previously isolated from 55 artisan dairy products from 12 locations in the Western Balkan countries (WBC) of Serbia, Croatia and Bosnia and Herzegovina. All strains were identified both by microbiological and molecular methods. The predominant species was *Enterococcus durans*, followed by *Enterococcus faecalis* and *Enterococcus faecium*. Over 44% of the isolates were resistant to ciprofloxacin and erythromycin, while 26.2% of the isolates were multi-resistant to three or more antibiotics belonging to different families. 185 isolates (29.1%) were susceptible to all 13 of the antibiotics tested. The antibiotic-susceptible isolates were further tested for possible virulence genes and the production of biogenic amines. Finally, five enterococci isolates were found to be antibiotic susceptible with good technological characteristics and without virulence traits or the ability to produce biogenic amines, making them possible candidates for biotechnological application as starter cultures in the dairy industry.

## Introduction

Bacteria of the genus *Enterococcus*, or enterococci, are considered lactic acid bacteria (LAB) (Schleifer and Ludwig, 1995). The usual ecological niche for *Enterococcus* species is the gastrointestinal tracts of humans and animals(Garg and Mital, 1991). However, enterococci are widely distributed in large numbers in foods, especially those of animal origin, such as various dairy and meat products, and some are also found in soil, water and on plants.

*Enterococcus faecium, Enterococcus faecalis*, and *Enterococcus durans* are the most prevalent species of enterococci in raw milk cheeses(Terzic-Vidojevic et al., 2007, 2009a,b, 2013, 2014a,b; Golić et al., 2013). They break down lactose and citrate during cheese ripening, which leads to the production of various volatile compounds, such as acetaldehyde, acetoin, diacetyl, and ethanol, which are responsible for the formation of the unique aroma and flavor of the final product (Andrighetto et al., 2001; Sarantinopoulos et al., 2001; Giraffa, 2002; Abeijón et al., 2006; Foulquié-Moreno et al., 2006). Due to their interesting metabolic and biotechnological traits (the ability to metabolize citrate, proteolytic and esterolytic activities, bacteriocin production, and their probiotic characteristics), enterococci may be utilized in food fermentation as commercial starter cultures(Tsakalidou et al., 1993; Centeno et al., 1999; Giraffa, 2003; Menéndez et al., 2004). On the other hand, enterococci have recently been found to be extremely important in clinical microbiology. Food-derived enterococci isolates are known to be resistant to a wide variety of antibiotics and to possess virulence genes (Giraffa, 2002; Saavedra et al., 2003; Veljović et al., 2014). These features contribute to the pathogenicity of enterococci, making them opportunistic pathogens. A major concern is the emergence of vancomycin-resistant enterococci (VRE), since this antibiotic is considered the last alternative for the treatment of multiple resistant infections (Teuber et al., 1999). Ideally, each strain that is intended to be used in a starter culture should be tested individually before any use in food or medicine, must not possess a single virulence factor and should be susceptible to relevant clinical antibiotics (Domig et al., 2003; Franz et al., 2003).

Due to their controversial status enterococci have been studied extensively (Galgano et al., 2001; Gelsomino et al., 2001; Morandi et al., 2006; Psoni et al., 2006; Gomes et al., 2008; Franz et al., 2011; Macedo et al., 2011; Ducková et al., 2014). Preliminary research on autochthonous LAB isolated from artisan dairy products of the Western Balkan region has indicated that about one third of all isolated LAB are *Enterococcus* species (Terzic-Vidojevic et al., 2007, 2009a,b, 2013, 2014a,b; Golić et al., 2013). However, a comprehensive study regarding their safe use as starter cultures in this region is lacking.

This paper evaluates the genetic diversity and antibiotic susceptibility of 636 *Enterococcus* spp. isolates from a laboratory collection including strains previously isolated from various artisan dairy products such as cheese, sour cream and kajmak from Serbia, Croatia and Bosnia and Herzegovina. The genetic diversity was explored by rep-PCR using the (GTG)_5_ primer, combined with 16S rDNA sequencing. In antibiotic susceptible strains the presence of virulence factors and production of biogenic amines were also determined. We aimed to provide the most complete information about the phenotypic and genotypic diversity, as well as the safety status, of natural enterococcal isolates from traditional dairy products of the Western Balkan region for their potential biotechnological application as starter cultures in the dairy industry.

## Materials and methods

### Bacterial strains, media, and growth conditions

All enterococci strains used in this study were isolated from 55 samples of different artisan dairy products collected from specific rural locations in Serbia, Bosnia and Herzegovina and Croatia in the period from 2003 to 2011. The samples of dairy products, together with their sources of isolation, are listed in Table 1. In total, 636 natural isolates of enterococci from the laboratory collection, initially identified by physiological tests, were used for the investigation of genetic and phenotypic diversity and antibiotic susceptibility.

**Table 1:** **The list of dairy samples used as source of enterococci**.

**No**.	**Sample of dairy product**	**Type of dairy product and number of enterococcal isolates**	**Region of dairy product sampling and references**
1	BGGO1	Cheese (8)	Serbia, Golija mountain;
2	BGGO2	Cheese (6)	(Terzic-Vidojevic et al., 2014a)
3	BGGO5	Cheese (6)	Serbia, Golija mountain; (Golić et al., 2013)
4	BGGO6	Cheese (6)	Serbia, Golija mountain; (Terzic-Vidojevic et al., 2014a)
5	BGGO7	Cheese (6)	Serbia, Golija mountain; (Golić et al., 2013)
6	BGGO8	Cheese (6)	Serbia, Golija mountain;
7	BGGO9	Cheese (8)	(Terzic-Vidojevic et al., 2014a)	
8	BGGO10	Cheese (8)	
9	BGGO11	Cheese (6)	Serbia, Golija mountain; (Golić et al., 2013)
10	BGVLJ1	Yogurt (1)	Serbia, Vlasina mountain lake;
11	BGVL1	Cheese (21)	unpublished data	
12	BGVL2	Cheese (2)	Serbia, Vlasina mountain lake;
13	BGVL2a	Cheese (15)	(Terzic-Vidojevic et al., 2013)	
14	BGPT1	Cheese (11)	Serbia, Stara Planina mountain;
15	BGPT2	Cheese (4)	(Begovic et al., 2011)
16	BGPT3	Cheese (3)	
17	BGPT4	Cheese (5)	
18	BGPT5	Cheese (34)	
19	BGPT6	Cheese (6)	Serbia, Stara Planina mountain;
20	BGPT7	Cheese (4)	unpublished data	
21	BGPT9	Cheese (9)	Serbia, Stara Planina mountain;
22	BGPT10	Cheese (11)	(Terzic-Vidojevic et al., 2009b)
23	BGAL1	Cheese (2)	Serbia, surrounding Aleksinac city;
24	BGAL2	Cheese (9)	(Golić et al., 2013)	
25	BGAL3	Cheese (12)	
26	BGLE1	Cheese (6)	Serbia, surrounding Leskovac city; (Golić et al., 2013)
27	BGBU1	Cheese (19)	Serbia, Beljanica mountain;
28	BGRE2	Cheese (15)	(Golić et al., 2013)
29	BGZLM1	Milk (10)	Serbia, Zlatar mountain;
30	BGZLS1	Cheese (3)	(Terzic-Vidojevic et al., 2007)
31	BGZLS10	Cheese (12)	
32	BGZLS20	Cheese (13)	
33	BGZLS30	Cheese (12)	
34	BGZLS45	Cheese (20)	
35	BGZLS60	Cheese (24)	
36	BGNV1	Cheese (23)	Serbia, Zlatar mountain; (Terzic-Vidojevic et al., 2009a)
37	BGTRS1	Cheese (33)	Bosnia and Herzegovina, Vlašić mountain;
38	BGTRS7	Cheese (5)	(Terzic-Vidojevic et al., 2014b)	
39	BGTRS10	Cheese (10)	
40	BGTRM1	Cream (25)	
41	BGTRM7	Cream (11)	
42	BGTRM10	Cream (14)	
43	BGTRK1	Kajmak (2)	
44	BGTRK4	Kajmak(2)	
45	BGTRK7	Kajmak(2)	
46	BGTRK10	Kajmak(11)	
47	BGPAS1	Cheese (26)	Bosnia and Herzegovina, surrounding Pale mountain city; unpublished data
48	ZGPR1	Cheese (15)	Croatia, Prigorje region; (Golić et al., 2013)
49	ZGPR2	Cheese (19)	
50	ZGPR3	Cheese (11)	
51	ZGBP4	Cheese (6)	Croatia, Bilogorsko-Podravska region;
52	ZGBP5	Cheese (19)	(Golić et al., 2013)	
53	ZGBP6	Cheese (18)	
54	ZGZA7	Cheese (19)	Croatia, Zagorje region; (Golić et al., 2013)
55	ZGZA9	Cheese (22)	

Enterococci and lactococci used as indicator strains for the analysis of antimicrobial activity of the enterococci strains were grown in M17 broth (Merck, GmbH, Darmstadt, Germany) supplemented with glucose (0.5% w/v) (GM17) at 30°C, while lactobacilli, used to screen the antimicrobial activity of *Enterococcus* sp. were incubated in MRS broth (Merck) at 30°C. Preliminary screening of 636 enterococci isolates for production of antimicrobial compounds was done by the deferred antagonism method using overnight cultures of isolates and various indicator strains. Briefly, soft GM17 and MRS agars (0.7% w/v) containing lactococci or lactobacilli indicator strains were overlaid onto GM17 and MRS plates, respectively. The plates were incubated overnight at the appropriate temperature (30 or 37°C) depending on the indicator strain. A clear zone of inhibition of indicator strain growth around the well was taken as a positive signal for production of antimicrobial compound.

*Escherichia coli* and *Staphylococcus aureus* were cultivated in Luria broth (LB), containing 0.5% NaCl, 0.5% yeast extract (Torlak, Belgrade, Serbia), and 1% bacto tryptone (Torlak) at 37°C while *Listeria innocua* was cultivated on BHI medium (Difco, Detroit, MI, USA) at 37°C. Corresponding agar plates were prepared by adding agar (1.7% w/v, Torlak) into each broth. The indicator strains used in this study for screening enterococci antimicrobial activity are listed in Table 2. The isolates were stored at −80°C in GM17 broth (Merck) supplemented with glycerol (15% v/v) and revitalized in the same medium by overnight growth at 30°C.

**Table 2:** **The list of strains used in this study for bacteriocin-activity detection**.

**Bacterial strains**	**Source**
*Lactobacillus plantarum* A112	Laboratory collection
*Lactobacillus casei* BGHN14	Laboratory collection
*Lactococcus lactis* subsp. *lactis* BGMN1-596	Laboratory collection
*Lactococcus lactis* subsp. *cremoris* NS1	Laboratory collection
*Lactococcus lactis* subsp. *lactis* biovar. *diacetylactis* S50	Laboratory collection
*Enterococcus faecalis* BG221	Laboratory collection
*Listeria innocua* ATCC 33090	ATCC^a^
*Escherichia coli* ATCC 25922	ATCC
*Staphylococcus aureus* ATCC 25923	ATCC

a *ATCC–American Type culture Collection, Manassas, VA, USA*.

### Bacterial identification

After microscopic examination, enterococci were identified to the genus level by Gram staining, catalase testing, arginine and bile-esculin hydrolysis, growth at 45°C and growth in NaCl 6.5% broth. All 636 enterococci isolates were subjected to the following tests: growth at 15°C, growth in broth with NaCl (8% w/v), production of CO_2_ from glucose, citrate utilization, acetoin and diacetyl production, time required for the formation of curd in reconstituted skim milk, exopolysaccharides (EPS) production, aggregation ability, and proteolytic and antimicrobial activity as described previously (Terzic-Vidojevic et al., 2007, 2009a,b, 2013, 2014a,b; Golić et al., 2013).

Identification of enterococci to the species level was performed according to Versalovic et al. (1994) using repetitive element palindromic-polymerase chain reaction (rep-PCR) analysis with (GTG)_5_ oligonucleotide (50-GTGGTGGTGGTGGTG-30). For this purpose the complete DNA from each of the 636 enterococci isolates was extracted as described by Hopwood et al. (1985) and details of the procedure has been reported by Terzic-Vidojevic et al. (2007). For sequencing of the 16S rRNA region, the complete DNA from certain enterococci isolates was used as a template for PCR amplification with UNI16SF (50-GAGAGTTTGATCCTGGC-30) and UNI16SR (50-AGG AGGTGATCCAGCCG-30) oligonucleotides (Jovcic et al., 2009). The PCR product obtained was purified by Qiagen (GmbH, Hilden, Germany) and sequenced (Macrogen, Amsterdam, the Netherlands and Seoul, South Korea). The BLAST algorithm was used to determine the most related sequences in the NCBI nucleotide sequence database (http://www.ncbi.nlm.nih.gov/BLAST).

### Antibiotic susceptibility testing

The antibiotic resistance of enterococci isolates was determined by the disc diffusion method recommended by the Clinical and Laboratory Standards Institute (CLSI, 2012). The following antimicrobial drugs (Bio-Rad, Marnes-la-Coquette, France) were used: vancomycin (30 μg), teicoplanin (30 μg), ampicillin, (10 μg), erythromycin (15 μg), tetracycline (30 μg), minocycline (30 μg), quinupristin–dalfopristin (15 μg), ciprofloxacin (5 μg), chloramphenicol (30 μg), nitrofurantoin (300 μg), and linezolid (30 μg), and for high-level resistance (HLR) gentamicin (120 μg) and streptomycin (300 μg).

### PCR detection of virulence determinants

The complete DNA of 11 antibiotic-susceptible enterococci strains with the best technological characteristics was used in PCR reactions to detect the presence or absence of genes for the following virulence determinants: cytolysin (c*ylA, cylB, and cylM*), aggregation factor (*agg*), gelatinase (*gelE*), enterococcal surface protein (*esp*), cell wall adhesions (*efaA*_*fs*_, and *efaA*_*fm*_), and sex-pheromones (*cpd, cob*, and *ccf*) according to Eaton and Gasson (2001), and collagen adhesin (*ace*) and hyaluronidase (*hyl*) as described by Vankerckhoven et al. (2004).

### Biogenic amines determination

The ability of the 11 chosen enterococci strains to produce biogenic amines was qualitatively determined on an improved screening medium as described by Bover-Cid and Holzapfel (1999) using four precursor amino acids: histidine, lysine, ornithine, and tyrosine.

### Statistical analysis

Classical ecology indexes were used to obtain species richness (S), with the Shannon–Wiener index (H′) indicating general biodiversity and Simpson's index (D) evaluating dominance of the species in each cheese sample, as follows:

$$\text{S}=\Sigma^{\text{N}};{\text{H}}^{\prime }=-\Sigma^{\text{N}}{\text{p}}_{\text{i}}\;{\text{log}}_{2}({\text{p}}_{\text{i}});\text{D}=1-\Sigma^{\text{N}}{\left({\text{p}}_{\text{i}}\right)}^{2}$$

Where N is the number of species and p_i_ is the number of isolates belonging to one species in the sample. Clustering was carried out in Statistica 7.0 for Windows (StatSoft Inc. USA) and in BioNumerics 6.5 using the algorithm “Unweighted Pair-Group Average Linkage Analysis.” Distances between the clusters were assessed using “Percent of disagreement.”

## Results

### The diversity of enterococci isolates

The general index of enterococci species diversity (H′) was calculated on the basis of the number of different enterococci species among the 636 isolates. The results showed that the dairy samples analyzed contained five *Enterococcus* species: *E. durans, E. faecalis, E. faecium, E. italicus*, and *E. avium*. The highest diversity and the lowest index of dominance of enterococci species were scored by samples from cheeses from the Prigorje region, Croatia (H′ = 1.61; D = 0.36) and Pirot region, Serbia (H′ = 1.5; D = 0.34), where four of five enterococci species were identified (*E. durans, E. faecalis, E. faecium, E. italicus*). In contrast, the lowest diversity (H′ = 0.99) and the highest dominance index (D = 0.62) were scored by samples from cheese manufactured in Pale, Bosnia and Herzegovina, where almost all isolates (20 out of 26) belonged to *E. faecium*. *E. durans* was the most abundant species in dairy products from Travnik, Bosnia and Herzegovina (84 out of 115), while *E. faecalis* was the most abundant in dairy samples from Zlatar Mountain, Serbia (84 out of 117). Interestingly, only 12 out of 636 isolates belonged to *E. italicus* and they were isolated from five out of 12 regions, while only one out of 636 isolates was identified as *E. avium* and was found in the Zlatar region, Serbia (Table 3).

**Table 3:** **The diversity of ***Enterococcus*** isolates from autochthonous dairy products collected at various geographic locations of the Western Balkan Countries**.

	***E. durans***	***E. faecalis***	***E. faecium***	***E. italicus***	***E. avium***	**Sum**	**H′**	***D***
Golija mountain	43	4	13	0	0	60	1.08	0.56
Vlasina lake	28	7	4	0	0	39	1.12	0.56
Pirot	41	21	21	4	0	87	1.5	0.34
Aleksinac	12	8	3	0	0	23	1.4	0.41
Leskovac	3	3	0	0	0	6	1	0.5
Beljanica	9	25	0	0	0	34	0.51	0.61
Zlatar mountain	15	84	15	2	1	117	1.26	0.55
Prigorje	15	21	8	1	0	45	1.61	0.36
Bilogorsko-Podravska	22	12	9	0	0	43	1.48	0.38
Zagorje	6	24	10	1	0	41	1.48	0.42
Travnik	84	16	11	4	0	115	1.22	0.56
Pale	2	4	20	0	0	26	0.99	0.62

### Genotypic characterization of enterococci

All 636 enterococci isolates were subjected to (GTG)_5_-fingerprint analysis. The results revealed that 278 out of 636 isolates (43.71%) differ among each other and have unique (GTG)_5_-fingerprint profiles, indicating great diversity of the isolates. Among them 124 isolates belonged to *E. durans*, 74 to *E. faecium*, 68 to *E. faecalis*, 11 to *E. italicus* and 1 to *E. avium* species. The fingerprint profiles obtained by (GTG)_5_-PCR of the enterococci isolates are presented in Figure S1. Interestingly, four main clusters have been determined. According to (GTG)_5_-fingerprint patterns, Cluster 1, comprising 46 isolates, includes the isolates with less genetic distance, mostly isolated from dairy products sampled in close geographical regions situated in the western part of Serbia. Cluster 2, comprising 124 isolates, includes more heterogeneous isolates, mostly isolated from dairy products sampled from southern Serbia, but also from Bosnia and Herzegovina. Cluster 3, comprising 10 isolates, includes only enterococci isolated from Bosnia and Herzegovina, specifically from milk and cheese sampled in the same household. Cluster 4 is the most heterogeneous according to (GTG)_5_-fingerprint profiles and encompasses the isolates originating from various geographical locations. It can be further divided into groups 4a and 4b. Group 4a is mostly comprised of the isolates originating from Golija Mountain, while group 4b is further divided to subgroups comprising isolates originating from western Serbia and Bosnia and Herzegovina. Hence, the results show that the corresponding groups of strains are partly correlated with the sources of isolation. Interestingly, a subdivision on the basis of households in the same locality was seen within the groups of isolates (e.g., the isolates from dairy products sampled in Croatia are scattered throughout the dendrogram), and isolates from the same households are divided among separate clusters. Finally, the isolates that were undistinguished by rep-PCR were classified on the basis of phenotypic characteristics.

### Phenotypic characterization of enterococci

Apart from general and genotypic diversity, the ultimate goal in exploring the diversity of natural isolates from various ecological niches is to determine their technological and functional potential. Hence, detailed phenotypic characterization of the enterococci strains with regard to acetoin (VP^+^) and diacetyl (D^+^) production, citrate utilization (C^+^), time of milk curdling (TMC), antimicrobial properties (Bac^+^), production of proteinases (Prt^+^), aggregation ability (Agg^+^), and EPS production was performed (Table 4). A large number of enterococci produced acetoin and diacetyl, 54.6 and 22.3% respectively. In addition, 40.9% of enterococci could utilize citrate as their only carbon source. On the other hand, only 4.2% of all the enterococci curdled milk within 6 h at 37°C (Table 4). Examination of the proteolytic activity of all 636 strains revealed that 17.5% of enterococci exhibited proteolytic activity as evaluated from β-casein hydrolysis by whole cells after 3 h of incubation. Almost 25% of all enterococci isolates showed the ability to produce antimicrobial compounds and most of those were enterococci that belonged to the *E. durans* species (29.3% of all 280 *E. durans* strains). Experiments with pronase E revealed the proteinaceous nature of the antimicrobial compounds, indicating that they could be bacteriocin-like substances (BLIS). Thirteen of 280 *E. durans*, three of 114 *E. faecium*, two of 229 *E. faecalis* strains and one *E. avium* strain had aggregation ability. Furthermore, one *E. durans* and one *E. italicus* strain were EPS producers. Some isolates exhibited two, three, or even four of the tested characteristics (Table 4).

**Table 4:** **Different technological activities in ***Enterococcus*** isolates from autochthonous dairy products**.

**Acetoin production (% of ent.)**	**Citrate utilization (% of ent.)**	**Diacetyl production (% of ent.)**	**TMC^a^ up to 6 h (% of ent.)**	**Bacteriocin production (% of ent.)**	**Proteinases production (% of ent.)**	**Aggregation ability (% of ent.)**	**EPS^b^ production (% of ent.)**
F2^c^	I	D	F1	D	I	A^g^	I
157/229 (68.6)	6/12 (50.0)	75/280 (26.8)	8/114 (7.0)	82/280 (29.3)	4/12 (33.3)	1/1 (100.0)	1/12 (8.3)
D^d^	F2	I	F2	F1	F1	D	D
138/280 (49.3)	113/229 (49.3)	3/12 (25.0)	15/29 (6.6)	31/114 (27.2)	21/114 (18.4)	13/280 (4.6)	1/280 (0.35)
F1^e^	D	F1	D	F2	F2	F1	F1
48/114 (42.1)	105/280 (37.5)	22/114 (19.3)	4/280 (1.4)	44/229 (19.2)	42/229 (18.3)	3/114 (2.6)	0/114 (0.0)
I^f^	F1	F2	I	I	D	F2	F2
3/12 (25.0)	36/114 (31.6)	42/229 (18.3)	0/12 (0.0)	1/12 (8.3)	44/280 (15.7)	2/229 (0.9)	0/229 (0.0)
A^g^	A	A	A	A	A	I	A
1/1 (100.0)	0/1 (0.0)	0/1 (0.0)	0/1 (0.0)	0/1 (0.0)	0/1 (0.0)	0/12 (0.0)	0/1 (0.0)
**A TOTAL**							
347/636 (54.6)	260/636 (40.9)	142/636 (22.3)	27/636 (4.2)	158/636 (24.8)	111/636 (17.5)	19/636 (2.98)	2/636 (0.31)

a Time of milk curdling,

b Exopolysaccharides,

c E. faecalis,

d E. durans,

e E. faecium,

f E. italicus,

g *E. avium*.

### Antibiotic susceptibility and resistance

An important issue in the selection of strains for safe use in food production is the characterisation of their antibiotic susceptibility in order to avoid uncontrolled spreading of the antibiotic resistance genes through horizontal gene transfer. Susceptibility of all 636 enterococci isolates was examined by the agar disc diffusion method using 13 antibiotics: vancomycin, teicoplanin, ampicillin, erythromycin, tetracycline, minocycline, quinupristin–dalfopristin, ciprofloxacin, chloramphenicol, nitrofurantoin, linezolid, gentamicin (HLR), and streptomycin (HLR) (Table 5). The results of antibiotic susceptibility revealed that a total of 451 out of 636 isolates (70.9%) were resistant to at least one of the tested antibiotics. One hundred eighty-two (28.6%) isolates were resistant to one antibiotic (151 to two, 77 to three, 26 isolates to four, 7 isolates to five, 4 isolates to six, 3 isolates to seven antibiotics, while 1 isolate was resistant to even eight antibiotics). It is worthwhile to note that the most resistant isolates belonged to *E. faecalis* species. In general, the highest percent of the enterococci isolates shown to have multiple resistances to various antibiotics originated from dairy products sampled from Pirot, Zlatar Mountain and Vlasina Lake in Serbia and the Bilogorsko-Podravski region and Zagorje, Croatia. On the other hand, a high number of the strains were susceptible to all tested antibiotics (185/636). Eighty nine of them belonged to *E. durans* species, originating mainly from Golija Mountain, Serbia and Travnik, Bosnia and Herzegovina. Thirteen of 31 antibiotic sensitive *E. faecalis* strains were isolated from the region of Zlatar Mountain, Serbia, while 11 of 28 and 9 of 28 antibiotic-sensitive *E. faecium* strains were isolated from the Pale and Travnik regions, Bosnia and Herzegovina. In addition, four strains of *E. italicus* species were also sensitive to all tested antibiotics (data not shown).

**Table 5:** **Regional distribution of antibiotic resistance of enterococci**.

**Region (number of enterococcal isolates)**	**Antibiotics (number of resistant isolates toward certain antibiotics)**												
	**VAN 30 μg**	**TEC 30 μg**	**AMP 10 μg**	**TET 30 μg**	**MNO 30 μg**	**ERY 15 μg**	**QDP 15 μg**	**CIP 5 μg**	**CHL 30 μg**	**HLG 120 μg**	**STR 300 μg**	**FTN 300 μg**	**LZD 30 μg**
**SERBIA**													
Golija (60)	0	0	0	3	0	18	0	12	0	0	0	22	3
Vlasina (39)	3	0	0	17	12	1	7	14	0	0	0	22	1
Pirot (87)	1	1	1	9	4	38	9	69	6	2	2	54	2
Aleksinac (23)	0	0	0	0	0	0	0	8	0	0	0	6	1
Leskovac (6)	1	0	0	4	0	0	0	5	0	0	0	2	1
Beljanica (34)	0	0	0	11	2	8	0	20	0	0	0	1	0
Zlatar (117)	23	0	0	22	9	9	13	73	0	2	2	9	30
Total Serbia (366)	28 (7.7%)	1 (0.3%)	1 (0.3%)	66 (18.0%)	27 (7.4%)	74 (20.2%)	29 (7.9%)	201 (54.6%)	6 (1.6%)	4 (1.1%)	4 (1.1%)	116 (31.7%)	38 (10.4%)
**CROATIA**													
Prigorje (45)	3	0	1	12	7	3	7	10	2	3	1	14	14
Bilogorsko Podravski region (43)	5	1	0	3	3	1	10	28	0	2	2	4	8
Zagorje (41)	4	0	0	14	2	5	25	21	0	0	0	3	13
Total Croatia (129)	12 (9.3%)	1 (0.8%)	1 (0.8%)	29 (22.5%)	12 (9.3%)	9 (6.97%)	42 (32.6%)	59 (45.7%)	2 (1.6%)	5 (3.8%)	3 (3.9%)	21 (16.3%)	35 (27.1%)
**BOSNIA AND HERZEGOVINA**													
Travnik (115)	0	0	1	16	2	2	3	12	1	0	2	11	8
Pale (26)	0	0	0	0	0	2	2	10	0	0	0	4	4
Total BH (141)	0	0	1 (0.7%)	16 (11.3%)	2 (1.4%)	4 (2.8%)	5 (3.5%)	22 (15.6%)	1 (0.7%)	0	2 (1.4%)	15 (10.6%)	12 (8.5%)
Total WBC (636)	40 (6.3%)	2 (0.3%)	3 (0.5%)	111 (17.5%)	41 (6.4%)	87 (13.7%)	76 (11.9%)	282 (44.3%)	9 (1.4%)	9 (1.4%)	9 (1.4%)	152 (23/9%)	85 (13.4%)

*VAN, vancomycin; TEC, teicoplanin; AMP, ampicillin; TET, tetracycline; MNO, minocycline; ERY, erythromycin; QDP, quinupristin–dalfopristin; CIP, ciprofloxacin; CHL, chloramphenicol; HLG, gentamicin; STR, streptomycin; FNT, nitrofurantoin; LZD, linezolid*.

### Virulence determinants and biogenic amines production

Finally, 136 out of 185 antibiotic-susceptible isolates, with unique (GTG)_5_-fingerprint profiles, were further analyzed in order to choose the candidates with the best technological properties. Based on the phenotypic characteristics, 10 *E. durans* strains [BGGO6-15 (D^+^, C^+^, VP^+^), BGAL3-19 (Bac^+^, C^+^, VP^±^), BGTRM1-52 (Bac^+^, Prt^+^, VP^+^, D^+^, TMC 6.5 h), BGTRM7-39 (Bac^+^, D^+^, C^+^, VP^±^), BGTRS1-10 (Bac^+^, Prt^+^, D^±^), BGTRS7-54 (Bac^+^, Prt^+^, VP^+^, TMC 6.5 h), BGTRS10-42 (Bac^+^, D^+^, VP^±^), BGTRK10-29 (C^+^, VP^+^, D^±^), BGPAS1-80 (VP^+^, TMC 6 h), and ZGPR2-1 (Bac^+^, D^+^, C^±^)] and one *E. italicus* [BGTRK4-42 (Bac^+^, D^+^, VP^±^)] were chosen.

The PCR analysis for detection of virulence genes (see PCR Detection of Virulence Determinants for details) revealed that none of the virulence genes was found in any of the analyzed strains. Moreover, the results obtained in this study indicate that five out of the 11 chosen enterococci strains (BGAL3-19, BGTRS7-54, BGTRM7-39, BGTRS10-42, and ZGPR2-1) were not able to produce biogenic amines in the presence of the precursor amino acids histidine, lysine, ornithine, and tyrosine (Table 6).

**Table 6:** **Production of biogenic amines in the presence of the precursor amino acids histidine, lysine, ornithine, and tyrosine**.

	**Histidine**	**Lysine**	**Ornithine**	**Tyrosine**
BGGO6-15	+	+	+	−
BGAL3-19	−	−	−	−
BGTRM1-52	−	−	+	−
BGTRM7-39	−	−	−	−
BGTRS1-10	−	−	+	−
BGTRS7-54	−	−	−	−
BGTRS10-42	−	−	−	−
BGTRK10-29	−	−	+	−
BGPAS1-80	−	+	+	−
ZGPR2-1	−	−	−	−
BGTRK4-42	+	−	−	−

## Discussion

Enterococci are found to be a normal part of the microbiota of artisan dairy products, especially in the Mediterranean region (Saavedra et al., 2003; Morandi et al., 2006; Psoni et al., 2006). However, their use as starter cultures in the dairy industry is still controversial since they have traditionally been considered indicators of fecal contamination. Moreover, enterococci are found to be involved in food spoilage (Franz et al., 1999) and food poisoning (Gardin et al., 2001), as well as in the spread of antibiotic resistance (Giraffa, 2002). Also, their role in the etiology of nosocomial infections cannot be neglected (Franz et al., 2003; Giraffa, 2003; Kayser, 2003). Hence, the aim of this paper was to evaluate the technological potential and safety issues for the use of natural dairy isolates of enterococci as starter cultures in the dairy industry. For that reason, in this study we have analyzed 636 natural isolates of *Enterococcus* sp. originating from various artisan dairy products collected in Western Balkan countries (WBC).

The results obtained in this study revealed the huge diversity among dairy enterococci isolates. The highest enterococci species diversity was recorded in fresh soft cheeses (1–10 days old) sampled from the Prigorje region, Croatia and Pirot, Serbia. Our previous results showed that enterococci and *Leuconostoc* sp. were the dominant species in the fresh soft cheeses of the Prigorje region, while lower diversity was found among other LAB species, indicating that enterococci were probably metabolically active and important for ripening of the artisan cheeses in that region (Golić et al., 2013).

Taking into account the diverse origins of the isolates, considerable genotypic heterogeneity was observed. The results showed that (GTG)_5_-fingerprint analysis was very effective in grouping and discriminating among enterococci strains, partly correlating with the sources of isolation. Hence, the results indicate that variable conditions, such as local vegetation or specific climates in the regions where dairy products are manufactured, significantly contribute to the diversity among enterococci strains. Interestingly, isolates belonging to the same species are found to be located in different clusters, indicating that identification by sequencing of 16S rDNA is not completely reliable in the case of natural isolates originating from complex communities such as dairy products. Possibly, horizontal transfer among bacteria living in the same ecological niche lead to their convergent/divergent evolution.

In order to explore the technological and functional potential of the natural enterococci isolates of artisan dairy origin, phenotypic variability was characterized. Due to various environmental pressures, natural isolates are usually shown to exhibit phenotypic variability (Giraffa et al., 2000, 2004). Enterococci play an important role in cheese ripening and contribute to the formation of the distinctive flavor of dairy products. Their ability to synthesize volatile compounds, such as diacetyl and acetoin, their proteolytic activity, and certainly antimicrobial activity are the important technological characteristics which make enterococci good candidates for starter and functional cultures for the dairy industry (Asteri et al., 2009; Nieto-Arribas et al., 2011). Enterococci usually exhibit weak proteolytic activities (Suzzi et al., 2000), and the best ability for casein degradation is shown by *E. faecalis* strains (Sarantinopoulos et al., 2001; Veljovic et al., 2009). Although the strains *E. faecalis* BGPT1-10P and BGPT1–78 were shown previously to have high activity in milk (Veljovic et al., 2009), in this study we found that only 17.5% of the analyzed strains exhibited proteolytic activity and it was equally distributed among *E. durans* (44/280), *E. faecium* (21/114), and *E. faecalis* (42/229) strains. According to the results of Morea et al. (1999) the degree of milk acidification by enterococci strains depends on the origin of the strain, hence it is highly likely that enterococci isolates of dairy origin are adapted to growth in milk. On the other hand, synthesis of diacetyl, acetoin from glucose, and citrate in the process of metabolic degradation by enterococci are shown to be very important for flavor formation in dairy products (Nieto-Arribas et al., 2011). Our analysis of enterococci dairy isolates from the WBC region showed that 347 out of 636 strains produced acetoin (54.6%), and 260/636 strains utilized citrate (40.9%), while 142/636 strains synthesized diacetyl (22.3%).

In addition, the excellent antibacterial activity shown by natural enterococci isolates make them promising candidates for food preservation and may contribute to the prevention of food spoilage (Viedma et al., 2009; Ananou et al., 2010). Bacteriocins produced by enterococci, enterocins, are very diverse and widely distributed among isolates. Production of enterocins in combination with a wide range of tolerance to high temperatures, dryness and increased salinity enables enterococci to become the dominant microbiota in fermented products (Franz et al., 1999). Our results support previous reports and indicate that the antimicrobial activity in natural dairy isolates from the WBC region showed a great effect on a number of pathogenic and non-pathogenic strains. Antimicrobial activity detected after treatment with protease suggests the proteinaceous nature of the bacteriocin activity. In our previous work it was shown that a number of *E. faecalis* strains exhibited an antimicrobial effect on *L. innocua* and *Listeria monocytogenes* (Veljovic et al., 2009). In particular, strains BGPT1-10P and BGPT1-78 showed antimicrobial activity against the Gram-negative strain *Pseudomonas* sp. PA17 and therefore could be used for the production of food biopreservatives (Veljovic et al., 2009). Apart from that, a number of *E. durans* and *E. faecium* strains exhibiting antimicrobial activity were found in this study.

Finally, with the aim of the safe use of enterococci as starter cultures in functional food, the frequency of virulence determinants and antibiotic resistance, as well as the synthesis of biogenic amines, was analyzed. The results showed that 185 out of 636 isolates (29.1%) were susceptible to the tested antibiotics, and as many as 59.6% of those isolates were resistant to two or more antibiotics. Interestingly, the presence of virulence determinants and antibiotic resistance is strain-dependent and region-specific. A high number of isolates from localities in Serbia (Zlatar Mountain, Pirot, and Vlasina Lake) had multiple antibiotic resistances. The results implicate the uncontrolled use of antibiotics in these regions, leading to antibiotic residues in feed or foods, which contribute to the occurrence of acquired and/or mutational antibiotic resistance mechanisms. More importantly, the further dissemination of antibiotic resistance genes among food-associated bacteria could cause the loss of natural isolates suitable for application in the dairy industry.

In order to eliminate the enterococci with pathogenic potential from among the possible candidates for use in dairy food production, and to avoid further transfer of virulence genes to other bacteria in the environment, identification of virulence factors is essential (Franz et al., 2003; Mannu et al., 2003). Fortunately, these important features are strain-specific, not species-specific. For this reason, each enterococcal strain intended for use in the dairy industry should be thoroughly tested for the absence of any pathogenic property. Ideally, a strain proposed for use in food production should not possess a single virulence factor and must be sensitive to relevant clinical antibiotics (Domig et al., 2003; Franz et al., 2003).

Virulence factors are commonly detected in clinical isolates of enterococci. Studies by Franz et al. (2001) and Eaton and Gasson (2001) have indicated the presence of virulence factors in food isolates, especially among strains of *E. faecalis*, which generally carry more virulence factors than strains of *E. faecium*. Our previous results showed that dairy strains *E. faecalis* BGPT1-10P and BGPT1-78 carry the *genE* gene and have gelatinase activity (Veljović et al., 2014). Moreover, in the strain BGPT1–10P a set of three genes of the cytolysin operon (*cylM, cylB*, and *cylA*) was detected, although two additional genes, *cylL1* and *cylL2*, required for the expression of hemolytic activity, were not identified in this strain (Veljović et al., 2014). The genes for hemolytic activity (*esp* and *efaA*) are found in high frequency in all tested *E. faecalis* strains (Veljović et al., 2014). However, in this study we demonstrated that none of the 11 selected *E. durans* dairy strains had any of the tested virulence genes, making them good candidates for use in the dairy industry.

Besides the analysis of virulence determinants and antibiotic resistance, an analysis of biogenic amine synthesis was performed. Biogenic amines are organic bases with aliphatic, aromatic, and heterocyclic structures produced through decarboxylation of the corresponding amino acid (Giraffa, 2002). The capacity for amino acid decarboxylation restricts the use of the strains in the dairy industry. The previous results of Valenzuela et al. (2009) showed that *E. faecalis* BGPT1-10P has the ability of tyrosine and ornithine decarboxylation. The strains tested in this study did not have the ability of amino acid decarboxylation.

## Conclusion

The results of this study reveal that autochthonous dairy products in the Western Balkan region are a rich source of new and diverse enterococci strains with considerable genetic, metabolic and technological potential. Since the role of enterococci in food spoilage and opportunistic infections is well known, before recommending the use of a particular strain as a starter culture it is necessary to characterize each strain in detail. This study reveals that out of 636 natural dairy enterococci isolates, only five strains belonging to *E. durans* have good technological potential and meet the safety criteria for use in the dairy industry. This finding points out the necessity for detailed characterization of enterococci isolated from dairy food (as a food of animal origin), since they could be reservoirs of antibiotic resistance and virulence genes, as well as producers of biogenic amines.

### Conflict of interest statement

The authors declare that the research was conducted in the absence of any commercial or financial relationships that could be construed as a potential conflict of interest.

## Supplementary material

Figure S1 **Dendrogram based on statistical analysis of the (GTG)_5_-PCR fingerprints of *Enterococcus* sp. isolated from autochthonous dairy products from Serbia, Bosnia and Herzegovina and Croatia.** The distances between the clusters were performed using “percent disagreement.” The algorithm “unweighted pair-group with average linkage” was used. The enterococci identified by 16S rDNA sequencing are given in bold.
