# Supplementary figures and images for: Diversity and antibiotic susceptibility of autochthonous dairy enterococci isolates: are they safe candidates for autochthonous starter cultures?
Source: Front Microbiol. 2015 Sep 9;6:954. doi: 10.3389/fmicb.2015.00954 (PMC4563272; doi:10.3389/fmicb.2015.00954)

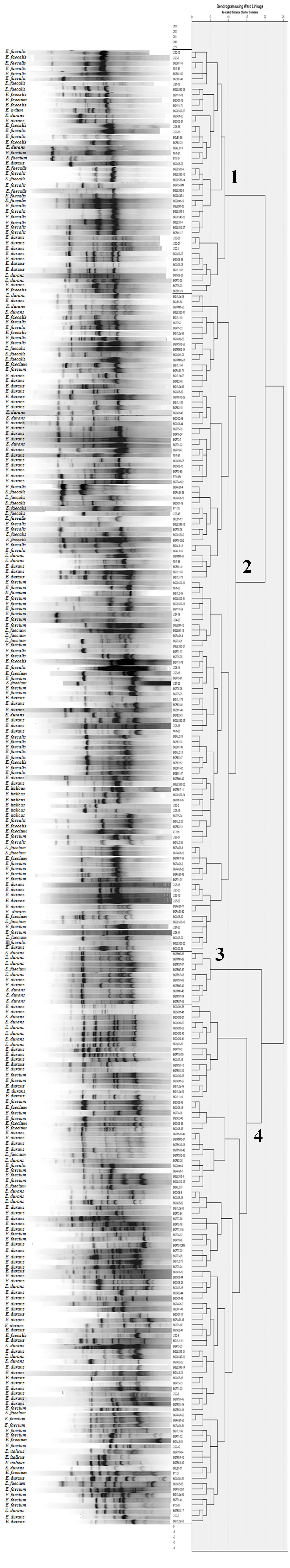

Supplement: Figure S1 — Dendrogram based on statistical analysis of the (GTG)5-PCR fingerprints of Enterococcus sp. isolated from autochthonous dairy products from Serbia, Bosnia and Herzegovina and Croatia. The distances between the clusters were performed using “percent disagreement.” The algorithm “unweighted pair-group with average linkage” was used. The enterococci identified by 16S rDNA sequencing are given in bold. [file Image1.JPEG]
